# Consequences of removing cheap, super-strength beer and cider: a qualitative study of a UK local alcohol availability intervention

**DOI:** 10.1136/bmjopen-2015-010759

**Published:** 2016-09-29

**Authors:** Elizabeth McGill, Dalya Marks, Colin Sumpter, Matt Egan

**Affiliations:** 1 NIHR School for Public Health Research (SPHR), London School of Hygiene & Tropical Medicine, London, UK; 2 Department of Social and Environmental Health Research, London School of Hygiene & Tropical Medicine, London, UK; 3 Camden and Islington Public Health, London Boroughs of Camden and Islington, London, UK

**Keywords:** alcohol, complex system, intervention, local policy, QUALITATIVE RESEARCH

## Abstract

**Objectives:**

Increasingly, English local authorities have encouraged the implementation of an intervention called ‘Reducing the Strength’ (RtS) whereby off-licences voluntarily stop selling inexpensive ‘super-strength’ (≥6.5% alcohol by volume (ABV)) beers and ciders. We conceptualised RtS as an event within a complex system in order to identify pathways by which the intervention may lead to intended and unintended consequences.

**Design:**

A qualitative study including a focus group and semistructured interviews.

**Setting:**

An inner-London local authority characterised by a high degree of residential mobility, high levels of social inequality and a large homeless population. Intervention piloted in three areas known for street drinking with a high alcohol outlet density.

**Participants:**

Alcohol service professionals, homeless hostel employees, street-based services managers and hostel dwelling homeless alcohol consumers (n=30).

**Results:**

Participants describe a range of potential substitution behaviours to circumvent alcohol availability restrictions including consuming different drinks, finding alternative shops, using drugs or committing crimes to purchase more expensive drinks. Service providers suggested the intervention delivered in this local authority missed opportunities to encourage engagement between the council, alcohol services, homeless hostels and off-licence stores. Some participants believed small-scale interventions such as RtS may facilitate new forms of engagement between public and private sector interests and contribute to long-term cultural changes around drinking, although they may also entrench the view that ‘problem drinking’ only occurs in certain population groups.

**Conclusions:**

RtS may have limited individual-level health impacts if the target populations remain willing and able to consume alternative means of intoxication as a substitute for super-strength products. However, RtS may also lead to wider system changes not directly related to the consumption of super-strengths and their assumed harms.

**Strengths and limitations of this study**

- This study uses a unique perspective by drawing on complexity theory to develop multilevel theories of change for an innovative alcohol availability intervention.
- Our qualitative methods lead to a pluralistic account of how the alcohol availability intervention may impact multiple outcomes and contexts.
- The study was conducted in a single English local authority, which allows for greater depth of analysis, but may limit the generalisability of the findings.
- The sample of hostel dwelling homeless people is relatively small but gives some of the most vulnerable and isolated community members a voice in the research.

## Introduction

Alcohol is a global health concern, a causal factor in over 200 diseases and conditions^1^ and contributes to healthcare costs,^2^ crime and disorder and losses of workplace productivity.^3^ Interventions that restrict the economic or physical availability of alcohol have been recommended to reduce alcohol-related harms.^4–7^ There is a pattern of research from different national settings supporting the case for national and mandatory interventions that restrict alcohol availability.^5^ Nonetheless, alcohol availability interventions are frequently delivered on a local and/or voluntary basis.^5^ ^8^ Reviews of alcohol availability interventions and health have found that the evidence base relating to local and voluntary initiatives is inconsistent and underdeveloped.^5^ This may be symptomatic of a broader perceived shortage of evidence to support public health decision-making relevant to local government and multisectorial initiatives.^9^

One recent UK alcohol intervention that embodies localist and voluntary characteristics is called ‘Reducing the Strength’ (RtS). With the encouragement of local authorities, shops licensed to sell alcohol for off-premise consumption (‘off-licenses’) voluntarily stop selling inexpensive high-strength (≥6.5% alcohol by volume (ABV)) beers and ciders, including products marketed as ‘super-strengths’ or ‘white ciders’. These products and their marketing have been said to encourage excessive drinking and harmful behaviours among vulnerable subpopulations.^10–12^ At the time of the intervention's implementation, a single 500 mL can of super-strength could exceed the (now former) UK health guidelines for daily alcohol consumption, while a single 3 L bottle of cider could exceed the weekly guidelines.^13^

RtS was first launched in Ipswich in 2012, and has been estimated to have been implemented in ∼80 local authorities across England,^14^ although some suggest this figure is too large, citing approximately 30 schemes in operation (personal correspondence with Robert Anderson-Weaver, Community Safety Officer, Portsmouth City Council, July 2016). Amongst RtS schemes, there has been some variation between areas with regard to the super-strength products targeted and linkages with services for the targeted populations.^15^ Guidance for implementing RtS identifies street and homeless drinkers as target populations^15^ based on assumptions about their consumption of these low-cost products, their vulnerability to alcohol addiction and perceived social problems around street drinking.^10^ ^16^ Numerous local studies of street drinkers and homelessness in the UK have pointed out that these are intersecting but not identical population subgroups.^17^ ^18^ Furthermore, homelessness can take different forms including rough sleeping, living in hostels, staying with friends and family, and often involves a residential instability that may lead to frequent changes in residential status.^19–22^

Alcohol availability modifications, such as RtS, are typically population-level interventions designed to encourage or compel changes in alcohol purchasing, consumption and health impacts.^4^ In the case of RtS, the physical and economic availability may be affected by the removal of cheap strong drinks from shops within a specific location. If many stores in a local area participate and remove super-strengths from their shelves, the variety of different types of alcohol available for purchase in that area may be reduced. The intervention also attempts to remove some of the very cheapest (measured as cost per unit of alcohol) beverages from the market, which would raise the price of the least expensive alcohol beverage available in participating shops. Even though the intervention itself may represent a relatively simple change to the local alcohol environment, the response of target populations and other agents within that environment is potentially complex.

Rickles, Hawe and others^23^ ^24^ have argued that neighbourhood and community interventions can often be considered ‘events’ in complex systems that may trigger chains of responses and relational changes between individuals or groups.^23^ ^25^ The complex system perspective argues that the most significant aspect of complexity lies not in the intervention itself, but in the system into which the intervention is introduced.^26^ Evaluating the impact of events within the system may involve monitoring how different agents within the system respond, considering intended and unintended consequences, and understanding how responses can potentially dampen or amplify the capacity of the intervention to contribute to system changes.^27^ ^28^ In this paper, we have conceptualised RtS as an event in a complex system.

This study explores how RtS was perceived and experienced by the target population of homeless drinkers and by service providers who work closely with this population. The aim is not to measure effects but rather to use a systems perspective to qualitatively explore how RtS may lead to intended and unintended consequences within the system in which it was implemented. For practical reasons, we have focused on hostel dwelling homeless people, acknowledging that this subgroup is associated with street drinking but still represents only one type of homelessness and one type of street drinker.^19–22^ We also focus on the views and experiences of service providers who work with those drinkers. We consider how both groups perceive the ways in which RtS may (or may not) influence their own activities, their peers' and the broader sociocultural environment that they inhabit.

## Methods

This study is part of a wider programme of research coproduced with local authority practitioners. An additional publication reports qualitative and quantitative findings relating to impacts on retailers and alcohol sales.^29^ The current study investigates the intervention from the perspective of a key target population, homeless people and service providers who work closely with that population. The research was conducted in mid-2014, after the intervention was implemented in late 2013. The study involved a focus group with alcohol service providers and interviews with alcohol service professionals, workers at homeless hostels, street-based services managers and hostel dwelling alcohol consumers (whom we refer to as ‘homeless’) (n=30). All participants were allocated a pseudonym.

Qualitative methods were considered appropriate for identifying a wide range of potentially relevant issues and providing opportunities for participants to introduce themes not considered at the research design stage.^30^ Evaluators have argued that qualitative research is particularly well suited to capturing the complexity of interventions and systems by unpacking processes by which interventions may trigger system changes.^31^ ^32^ This complexity may include multiple and unanticipated outcomes over variable time frames, competing aims and values of stakeholders and target populations and non-linear relationships between contexts, processes and outcomes.^23^ Qualitative approaches that do not explicitly incorporate a systems lens may still include some or all of these features, but a systems approach encourages a framework for analysis that explicitly focuses on changes to behaviours and relationships between agents at multiple levels in response to an intervention.^23^ ^28^ The flexibility of qualitative methodologies can also help researchers overcome some of the barriers to evaluating local health policy innovation, which can include small delivery scales, rapid delivery timescales^33^ and a demand from local decision makers for evidence that is sufficiently contextually rich to be recognisable to them as ‘local’.^9^ ^34^

### Intervention and setting

The study focused on an inner-London borough characterised by high population density, social inequality and a high degree of residential mobility. In late 2013, off-licence shops in three ‘hot spots’ for street drinking were asked to voluntarily stop selling super-strength products. Local authority data showed these areas to have a very high alcohol outlet density and alcohol retailers in these areas primarily consist of small, independent ‘newsagent’ stores who open late and rely on alcohol as a large proportion of their total revenue. According to a local authority audit, super-strength products were often, although not always, the cheapest alcohol products available for purchase in these stores. The RtS intervention was planned and implemented by the borough's council and police licensing teams and supported by community safety officers. The intervention has five stated aims, which are presented in box 1.

Box 1 **RtS aims in one English local authority**

1. To remove ‘super-strength’ from off-licences;

2. Voluntary variation of existing licences to include a condition not to sell ‘super-strength’;

3. To reduce crime and antisocial behaviour (specifically street drinking and begging);

4. To reduce alcohol-specific admissions including repeat admissions;

5. To highlight the dangers of alcohol, particularly super-strength alcohol, to residents.

Prior to the intervention, 39% of the 78 off-licenses in the RtS area sold super-strength products. Following the intervention launch event, implementers reported that all but two off-licences agreed to participate in the scheme. At 6-month follow-up, implementers reported around 95% of off-licences continued to participate and considered this a substantial reduction in super-strength availability for those areas.

### Recruitment and data collection

Homeless people are recognised as vulnerable and isolated groups, raising ethical and practical issues affecting recruitment and data collection. Service providers were interviewed to draw on their knowledge of homeless drinking behaviours but also to allow identification of contrasting perspectives between the two groups of participants. Participants were recruited through stakeholder contacts and direct approaches to hostels and services. Homeless participants received information about the study from service providers with an invitation, but no obligation to take part. The mediating role of the service providers meant we were unable to track participants (homeless or otherwise) that were informed of the study but declined to take part. Participants all received an information sheet and verbal information about the study; all recruitment was based on voluntary informed consent.

Most of the fieldwork involved semistructured individual interviews conducted by EM (a research fellow with prior experience of interviews, focus groups and qualitative analysis); each participant was interviewed once. Service providers were not present when homeless participants were interviewed, and participants were not interviewed in front of their peers. Some alcohol service professionals requested a focus group for logistical and time management reasons. Service provider topic guides included sections on alcohol and homeless service provision, homeless people's drinking behaviours and the RtS intervention. Drinker topic guides covered similar themes but focused more on the participants' own behaviours and experiences. We asked specifically about super-strength consumption, but also more generally about how drinkers would respond to restricted alcohol availability. Interviews were conducted in a private area in work settings or hostels, audio recorded and transcribed. The researcher also made field notes during and after each interview. Homeless participants received a £10 voucher as compensation for their time.

### Analysis

A total of 723 min of audio was recorded and transcribed; this figure excludes tours around five homeless hostels during which participants provided the researcher with background information. The first author coded the transcripts in NVivo V.10 using the interview guide to group major themes; a second researcher double-checked the coding. We then used concepts from complexity theory to deductively code the transcripts. Specifically, we have used participant perspectives to identify theories of change—including participants' views on what constitutes potential intended and unintended consequences that could follow from the implementation of RtS.

## Results

In total, 30 people participated in the study (table 1). The nine alcohol consuming hostel residents were predominantly male and seven had been in the hostel system for over a year. Six reported previous experience of rough sleeping. Four stated that they were regular (daily) consumers of super-strengths while others consumed it less frequently, preferring alternatives such as wine, vodka, or regular beer and cider. A total of 21 service professionals participated in the study, 11 in a focus group at an alcohol service centre and 10 individual semistructured interviews were conducted with professionals in other services.

**Table 1:** Number of participants

	Individual interview	Focus group	Males	Females
Participants				
Homeless drinkers	9	0	8	1
Alcohol service managers and staff	2	11	4	9
Hostel managers and staff	6	0	2	4
Street-based services managers	2	0	2	0

**Figure 1:**
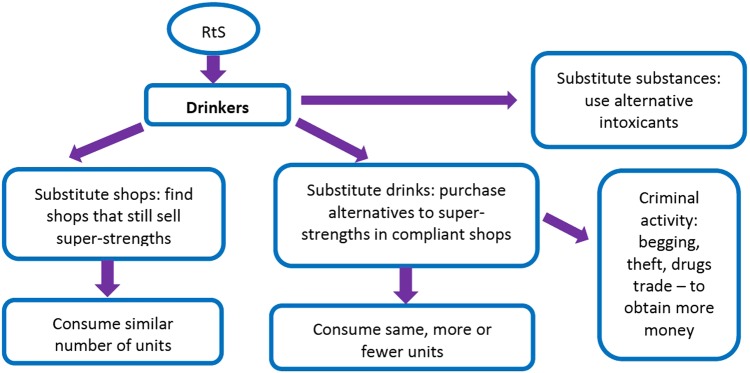
Individual level theories of change.

**Figure 2:**
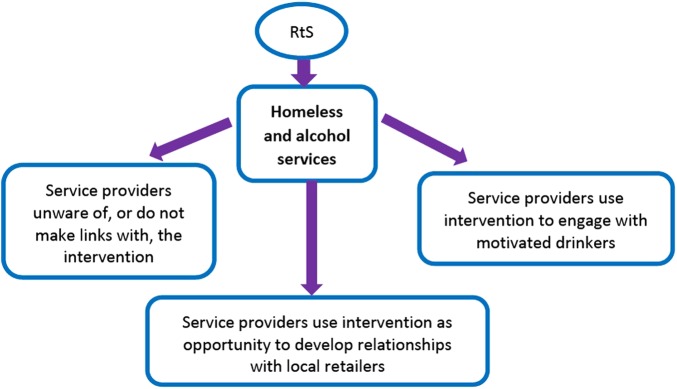
Service level theories of change.

Using participant perspectives, we structured our analysis to consider different levels or domains at which the intervention constitutes an ‘event’ and where participants saw potential impacts stemming from the implementation of RtS. This includes the levels of the individual and service provision, as well as potential broader sociocultural implications. The levels of the individual drinker (figure 1) and service provision (figure 2) were inherently built into our sampling strategy, whereas the broader sociocultural context emerged from participants' accounts.

### Findings at the individual level

Homeless drinkers and service providers presented a range of opinions about which groups they thought the intervention targeted, which included but was not limited to street drinkers, rough sleepers and hostel residents. More broadly, participants tended to assume that super-strength products were consumed by disadvantaged, middle-aged males with high levels of alcohol dependency described by various service providers as ‘problematic’, ‘physically dependent’ or ‘hard-core entrenched’ drinkers.

Drinkers, and some service providers, had noticed the reduction of super-strength availability within the intervention areas and explained that only a limited number of shops continued to sell the products:

I don't know if you're aware of that as well, but you know the strong lagers, i.e. the Special Brew and the Skol Super Light, all the 24 hours shops around here, all the police have completely stopped them from selling it, you can't buy any strong beers anywhere around here anymore. You know, except for a very select couple. (Christopher, drinker)

now the Reduce the Strength campaign is in effect so a lot of these are no longer selling those brands that I just mentioned. However, there are still one or two doing it. (Luke, street-outreach manager)

Participants discussed this substantial, but not absolute, restriction in super-strength availability as an event that could lead to a number of substitution responses. Drinkers described still being able to purchase super-strengths by switching from compliant to non-compliant shops. For example, Timothy described how super-strength drinkers walk a greater distance to find stores that continue to sell super-strengths:

That's what everyone does at the minute, they walk out further afield to get it…they go into the shops that still do sell it, which is only like a handful, not even a handful, a couple of them. (Timothy, drinker)

Drinkers disagreed about whether the necessity of walking longer distances would affect their purchasing behaviour. One said ‘I'll walk as far as I can to get my same beer’, (Max, drinker) whereas others suggested there was a limit to the distance they would walk and this might vary depending on time of day. Service providers also reported seeing homeless and street drinkers, and alcohol-service clients still consuming super-strength products.

A second substitution behaviour participants described was substituting drinks within compliant shops. Without prompting, several drinkers attempted to calculate the ways they could continue to consume the same number of units of alcohol within stores participating in RtS. Some suggested they would switch to drinks with higher alcohol contents, such as wine, sherry or vodka. For example, Christopher, a super-strength drinker, described how drinkers can still purchase vodka at compliant stores:

You can't buy any strong beers anywhere around here anymore, except for a very select couple, but it hasn't deterred anyone though has it? Christ, yeah, cos they've still got bottles of vodka in there. (Christopher, drinker)

Other drinkers and service providers, however, questioned whether many homeless drinkers would be able to budget for the higher cost of a larger bottle of spirits (which were assumed to represent better value than smaller bottles) or make a bottle last longer than a day.

Service providers also hypothesised that if a sufficient number of stores participated in the intervention, thus resulting in an absolute reduction in the availability of super-strengths, drinkers might purchase greater quantities of cheaper, weaker beer or cider. However, drinkers largely rejected this idea as they perceived such drinks to be insufficiently strong to achieve a feeling of intoxication, or prevent withdrawal symptoms. One drinker called ‘normal’ strength beers ‘a waste of time’ (Christopher) and another described them as ‘piss water’ (Joshua).

Several drinkers and service providers also suggested that more drinkers would engage in alternative substance abuse, as many had histories of codependency. This could include illegal drugs or products not intended for consumption, such as cleaning products or solvents:

So I have one beer or one [butane] gas, but what I worry about there is once I've finished that beer, then I've probably by that time nearly gone through half of that one gas…When I really am getting anxiety attacks from the alcohol comedown and all that kind of stuff, the gas really douses it, you know? (Christopher, drinker)

I think the people who need alcohol and haven't got any money…can do extreme things [such as] drink a hand sanitizer in hospitals…I think it's a least bad thing if people can drink something that's at least commercially produced and safe. (Lauren, alcohol service professional)

Participants acknowledged that purchasing more expensive drinks or alternative substances could result in unintended consequences for drinkers and perhaps the broader community should drinkers turn to crime or begging to obtain these products. One super-strength drinker, who distanced himself from these behaviours, argued that other homeless drinkers would ‘try and blag or steal, or whatever it takes, you know to get it, as I said, it won't make much difference’. (Kevin, drinker). Service providers also considered these possibilities, arguing:

I think the other thing that would happen is that you could see offending go up. (Lauren, alcohol service professional)

If the money's not there they might turn to committing crime. (William, alcohol service manager)

On the other hand, a hostel employee argued that any potential spike in more visible or risky forms of crime would only be short lived:

In terms of sustainability it probably depends on the risk associated with whatever they're doing. So things like pickpocketing is quite high risk because you're quite likely to attract the attention of the police and so that's probably not sustainable. (Peter, hostel staff)

### Findings at the service level

Within the complex system in which RtS is implemented, there are also consequences for service provision (figure 2). The integration of RtS with existing homeless or alcohol services was a particular concern for service providers who largely saw the intervention as too limited to effectively address excessive alcohol consumption. Several participants attempted to reframe the problem away from alcohol availability and instead emphasised either psychological problems or wider social ‘causes’ of alcohol misuse such as poverty and homelessness:

I don't think [RtS] acknowledges the psychological reasons why people drink, I don't think it acknowledges all the kind of needs that are being met, albeit in a maladaptive way by alcohol. (Adam, hostel manager)

Service providers who were sceptical about the potential benefit of RtS did note that it might be used as a tool to engage drinkers who were already seeking help. For example, the intervention could be used to help talk to their drinkers about reducing alcohol consumption in conjunction with support plans:

it helps us because you could in your harm minimisation support plans say drink at different times, drink a lower strength beer, drink less amount and only go to that shop.…if you know they're not selling strong drinks you can make it all part of the task-oriented support plan. (Thomas, hostel manager)

Service providers tended to agree that in this particular roll-out of RtS, there was a missed opportunity for public services, including the alcohol services, hostel services and the council, to engage and interact more closely with the business sector. Some of these service providers had not heard of RtS and felt that explicit links between different stakeholders could have initiated positive changes. If implemented to encourage service linkage, RtS was seen as an opportunity to work more closely with local shop managers to assist dependent drinkers through alcohol supply regulation:

I can't understand why we [the alcohol service] weren't asked to participate because we have a lot of volunteers and services that would have been able to contribute by going around to some of the shops as well because I think it's been about trying to get the shop owners to take responsibility for the community. (Eleanor, alcohol service professional)

### RtS within the wider sociocultural environment

Participants also described how RtS may have implications beyond individual drinkers and service provision for homeless drinkers. Specifically, participants situated RtS within a broader sociocultural context, of which they are a part, and described how the intervention may influence social norms around drinking. Participants also considered, as individuals targeted by RtS and service providers working with that population, the ethics of social policies, such as RtS, that target specific groups of individuals.

#### Social change: making alcohol the new tobacco

Service providers positioned the intervention within the broader culture of drinking in England. The participants argued that even if RtS had little immediate impact on local drinking behaviour, it might still contribute to a long-term process of social change and public awareness around alcohol-related harms. One hostel manager said that RtS could be ‘part of a whole move of this awareness of how dangerous drink is. So I think it will have an effect but I think it's going to be part of a long term social change. I think in the short term it's going to be very patchy’. (Thomas, hostel manager)

Several providers drew on the history of tobacco and argued that political action and interventions around smoking ultimately changed cultures around smoking, particularly around the public acceptability of smoking in public. Service providers saw parallels between tobacco policy and RtS:

…and then the culture has changed as well…because the first place that implemented no smoking in public places was California and I think at the time in England the general perception was it was almost like a communist style, sort of undemocratic thing that would be unimaginable…[It] was a shock but then the culture changed and actually now everyone just thinks it's the norm. (Patrick, alcohol service professional)

#### Ethical considerations of targeted policies

Service providers and drinkers believed RtS contributed to a broader strategy of targeting disadvantaged populations. Several service providers justified this targeting on the grounds that people who consume super-strength disproportionately use public services, cause antisocial behaviour and are vulnerable to environmental health risks:

…people that are actually dying or you know been affecting the community in a big way, I think those are the specific target groups that they're looking at. Those people that are actually impacting on the community, causing a lot of disruption, causing a lot of offending. (Jessica, hostel manager)

Among the drinkers, there was confusion surrounding why super-strength drinks were targeted when other drinks such as spirits or wine have higher alcohol contents. Several homeless participants had the view that targeting the most disadvantaged with availability restrictions was a social injustice, and one hostel manager expressed concerns about how alcohol-related harms among more affluent members of the population were not addressed by the intervention:

It's a bit unfair…the middle, upper class [have their] nose up in the air with a nice glass of claret or a glass of rosé or whatever, they drink as much as I do. So, please do not tell me I'm the only alcoholic. (Kevin, drinker)

some people could argue it could be a bit of a class sort of thing really demonising poor people. (Nathan, hostel manager)

## Discussion

We have conducted qualitative research to obtain different stakeholder perspectives on the potential impacts of RtS following its implementation in a London borough. We have deliberately constructed a pluralistic account based on the understanding that the intervention is an event in a complex system. RtS is assumed to make positive and negative contributions in advancing health and social policy goals relating to reducing alcohol harms.

Participants suggested that at the individual level, the target population were likely to adopt substitution behaviours to seek to reduce the impact of the intervention on their intoxication. Such adaptations could involve finding stores still selling super-strengths or continuing to shop at participating stores and substituting drinks, including drinks with higher prices. Recent research on dependent drinkers' purchasing behaviour in Scotland found drinkers seek the cheapest alcohol beverages from their local stores and adapt their purchasing behaviour based on price, the alcohol environment and drink preferences. The authors conclude that ‘heavy drinkers are astute, skilled and flexible shoppers’ (ref. 35, p. 1578). Our findings on substitution behaviours in response to RtS corroborate these conclusions. Participants also suggested, with some differences of opinion, responses around illicit drug and substance abuse, or crime and antisocial behaviours that could potentially affect individuals, retailers and communities.

At the service level, we found different viewpoints about how successfully the intervention had linked with other services. Some participants felt the intervention, as delivered in this local authority, had missed opportunities for service providers to engage with a range of stakeholders. However, some participants believed that RtS could offer opportunities for public and private sector stakeholders to strengthen or modify relationships in order to further encourage joined-up services to tackle deeply entrenched alcohol problems.

Participants also contextualised the intervention within a broader sociocultural environment and, as members of that culture, suggested how RtS may lead to broader cultural changes. Drawing on the history of tobacco policymaking, some participants suggested that local initiatives, such as RtS, could be a contributor to cultural changes surrounding the acceptability of harmful alcohol consumption. From this perspective, small interventions were considered to be important as part of a cumulative escalation of action and debate around alcohol: a different kind of impact to that normally considered by intervention effectiveness evaluations. As further evidence of this ‘escalation’, the Portman Group, a UK association funded by the alcohol industry, recently issued guidance discouraging the sale of single cans of super-strengths that exceed daily drinking guidelines for men and women.^36–38^ However, drinkers and service providers in this study highlighted how the highly targeted product restriction ignored other more commonly consumed alcohol products, and the problems of excessive drinking that exist across the whole population. Policies such as RtS may be seen as indicative of cultural associations of ‘problem drinking’ with more marginalised populations.

Findings from our study add to a small body of research on highly targeted alcohol availability interventions. For example, in remote Australian communities, where the sale of cask wine in containers over 4 L was banned, mixed methods evaluations found that while there was significant substitution, either to other drinks or to other localities, that there was still an overall reduction in alcohol consumption not entirely offset by the substitution.^39–41^ A UK study exploring public acceptability of policies to reduce alcohol consumption found participants repeatedly attempted to reframe problems related to alcohol availability in favour of a broader perspective that links alcohol harms with social and cultural characteristics and values.^42^ Similar reframings can be found in some of the comments made by participants in this study. A related study found evidence of public concern that people who are sufficiently motivated will circumvent interventions,^43^ a process which may encourage uptake of additional risky behaviours.^42^ Our findings on individual-level responses corroborate these findings.

### Strengths and limitations

For pragamtic reasons, we interviewed homeless alcohol consuming individuals who reside in hostels but recognise that other groups, such as rough sleepers and independent-living super-strength consumers, are also affected by the intervention. Our participants already engage, to varying degrees, with some services, by virtue of living within the hostel system. Drinkers who live independently, or are disengaged from services, may have provided different accounts of how they experienced the intervention. Informal discussions with implementers revealed that they felt they did engage with a range of alcohol and homeless services, whereas our findings from the service providers provide a different view. Future work could fruitfully bring together these perspectives.

We used a single case study site. The choice between a single or comparative case study is to some extent a trade-off between depth of analysis in a single site and greater breadth that may result from multiple sites. Our sample, though small, was sufficient for us to generate multiple theorised pathways to impact including substitution behaviours and other responses to RtS which, we believe, can be plausibly considered by practitioners in other settings. We may speculate as to whether or not our findings covered all possible pathways (and so claim data saturation), but we have no clear way of determining this. Those pathways we did identify tended to recur in multiple interviews and gave us confidence that we had identified responses that appear particularly relevant for theorising potential impacts.

Some of the participants' responses were grounded in direct personal experience, but some less so. Although the intervention achieved high levels of compliance from shops, participants reported being able to continue purchasing super-strength products with relative ease. While this was itself an important finding, we also asked participants about their hypothetical responses, should RtS be implemented by all local shops. It might be assumed that when participants' responses are grounded in their experience, this may constitute more powerful evidence than the speculative responses, although both shed light on how they perceive the intervention—in its current form and in a hypothetical more full realised form—and both are subject to potential biases or may be interpreted as telling us more about how people represent themselves than how they actually behave.^44^

We have used interviews and a focus group to obtain participant perspectives on intended and unintended consequences following the implementation of RtS. Given the sensitive nature of the topic and some of the behaviours we asked about, there is a potential for social desirability bias. While we recognise this as a limitation that may have been addressed through the use of ethnographic methods, we also note that participants spoke openly about their experiences and behaviour, at times presenting themselves in a ‘negative’ light.

While our study identified different types of substitution behaviours that could potentially be used to circumvent the intervention, additional qualitative and quantitative research is required to measure the extent to which different types of substitution occurred.

## Conclusions

The use of qualitative research methods has allowed us to create a pluralistic account of how RtS may affect the components of the system in which it is implemented, and has illustrated the mechanisms by which such changes may occur. We argue that the small scale of implementation and the limited range of products affected make it plausible that RtS could, by itself, make only a modest impact on alcohol harms. We base this on the apparent ease and willingness of drinkers to use substitution behaviours, including switching shops, drinks or substances in order to circumvent the availability restrictions. These individual responses are reactions to the physical and economic dimensions of alcohol availability. An approach that ensured full shop compliance across larger geographical scales could restrict drinkers' ability to substitute to non-compliant shops. Hence, we hypothesise that the local and voluntary nature of RtS could be barriers to effectiveness, although a well-conducted quantitative evaluation is required to test this.

However, our systems approach has also encouraged us to consider effects on services as well as effects on individual drinkers. Although RtS in this local authority was seen as a ‘missed opportunity’ for service providers to engage with a range of stakeholders, some front line staff believed that RtS has the ability to facilitate new forms of engagement between public and private sector interests and promote further awareness of alcohol harms. Hence, some stakeholders suggest that a small, local intervention, such as RtS, can potentially contribute to wider system changes irrespective of, or indirectly related to, the intervention's effectiveness in achieving its formally stated goals.
